# All camouflage strategies are not equal

**DOI:** 10.1098/rspb.2022.1869

**Published:** 2022-11-30

**Authors:** Amanda M. Franklin

**Affiliations:** School of BioSciences, The University of Melbourne, Parkville, VIC 3010, Australia

The difference between life and death can come down to only a couple of seconds or a few millimetres. An animal could avoid being eaten if it can remain undetected for a few extra seconds or cause a predator to misjudge its attack by a few millimetres. Camouflage plays a crucial role in providing these precious seconds or millimetres, but are all camouflage strategies equally effective? de Alcantara Viana *et al*. [1] examine the efficiency of different camouflage strategies by conducting a thorough meta-analysis. They compare predator search time and attack rate among five different camouflage strategies, three predators and three prey types, showing that both camouflage strategy and prey type can influence effectiveness. Their study provides a valuable synthesis of the current research and highlights several key directions for future studies.

Camouflage strategies have evolved in response to multiple selection pressures and vary in their benefits and costs. Some strategies are excellent to avoid detection but ineffective when the prey moves, whereas other strategies help moving prey to evade an attack but have limited effect on reducing detection or recognition. de Alcantara Viana *et al*. [1] provide support for these differing benefits and costs, demonstrating that strategies that prevent prey detection (background matching, disruptive coloration) or prey recognition (masquerade) increase predator search time, whereas strategies that deflect attack or reduce accuracy (eyespots, motion dazzle) do not impact search time. They also found that the three strategies that reduce attack rate—background matching, disruptive coloration and motion dazzle—cause a reduction of similar magnitude. This suggests that avoiding detection or recognition in the first place is most beneficial.

The effect of motion dazzle on reducing attack rate may not be fully represented by this meta-analysis due to limited research. Only 17% of studies in the meta-analysis studied this defence, compared to 38% for background matching and 26% for disruptive camouflage. This scarcity of research is likely because it is difficult to capture the complexities of motion dazzle. Motion dazzle requires the movement of both prey and predator, and is also likely to depend on background complexity, background movement and lighting environment. Few studies have tackled this camouflage tactic, and it is a significant gap in our understanding of camouflage strategies. Emerging technologies, such as computer graphics, have allowed researchers to begin to delve into contexts and prey characteristics that influence the success of motion dazzle. These studies demonstrate that effectiveness can depend on prey size, pattern contrast, movement trajectory, speed of movement and background complexity [2–4]. To build on these studies, more research with realistic scenarios and a variety of different predators is required to fully appreciate contexts in which motion dazzle is beneficial.

Visual systems can vary dramatically among predators. However, the authors found no difference in search time or attack rate among birds, fish and humans. This supports previous work demonstrating that humans and birds have fundamental similarities in visual processing and use similar features for object detection and recognition [5,6]. Many studies leverage these similarities and use human participants to answer questions that are difficult or impossible to address with animals (e.g. does a camouflage strategy influence detection or recognition?). However, vertebrates share fundamental similarities in eye design, such as possessing two camera eyes and similar photopigments, that can be very different to invertebrates [7]. Visual systems of invertebrate predators, such as dragonflies or jumping spiders, are not only very different to vertebrates, but often differ greatly from one another. Therefore, camouflage strategies may not be effective against all predators or how the strategy works may vary among predators. Understanding how a variety of predators perceive different camouflage strategies is necessary to understand the mechanism behind how these strategies function and the selective pressures influencing the evolution of camouflage strategies.

The generality of conclusions from meta-analyses is necessarily constrained by the data they draw from. The studies accumulated by de Alcantara Viana *et al*. [1] were largely from Europe and the UK, with few in tropical locations or other continents. Habitats and predator communities, however, can vary dramatically across continents, between tropical and temperate locations and between aquatic and terrestrial systems. For example, tropical rainforests often have greater background complexity, glossier backgrounds and different lighting environments compared to temperate forests. Background structural complexity and lighting conditions can influence the survival of camouflaged prey [2,8,9]. It is possible that the efficacy of different camouflage strategies varies in different environments, but more research is required to fully appreciate environmental drivers of camouflage.

Camouflage research is moving in fascinating new directions with studies beginning to assess changeable prey colorations, dynamic visual scenes and the visual capabilities of different predators. Iridescence, a visual effect in which colour depends on viewing geometry, has recently been shown to improve the survival of prey [8]. Furthermore, improved survival is greater for prey on glossier leaves, demonstrating that changeable visual effects associated with the prey or background can influence camouflage. Dynamic lighting environments, such as dappled light or water caustics, can also improve prey survival by increasing noise in the visual scene (figure 1) [9]. Intriguingly, recent research shows that polarization vision can help to counter the effects of a dynamic lighting environment for predators [10]. This highlights the importance of investigating different predators and visual system processes. Together, these examples show an exciting trend towards assessing more realistic and complex camouflage strategies and environments, shedding light on different drivers and functions of camouflage and animal coloration.

**Figure 1.**
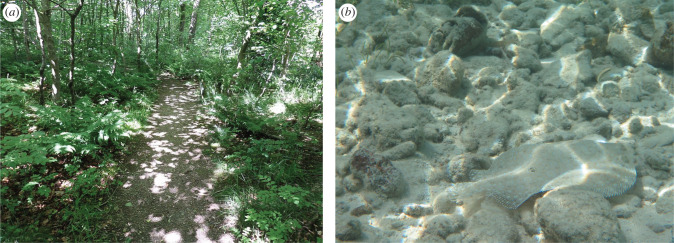
Dynamic lighting environments, such as dappled light (*a*) or water caustics (*b*), can influence camouflage effectiveness. Photos: (*a*) Rosser1954 WikiMedia (CC BY-SA 4.0); (*b*) Amanda Franklin. (Online version in colour.)

de Alcantara Viana *et al*. [1] contribute a thorough synthesis and discussion to the rapidly expanding literature on animal camouflage strategies. Of particular importance, their literature search highlights key gaps that require attention, including expanding studies to different predators, habitats and contexts. In combination with recent efforts to investigate more complex and dynamic scenarios [8–10], additional studies are likely to lead to exciting discoveries about new camouflage types and will also contribute to identifying generalities across different scenarios or context dependence of camouflage strategy effectiveness. This could have implications for diverse fields including evolutionary biology, conservation, military camouflage or computer vision. The challenge will be to link the effectiveness of different camouflage strategies with dynamic environmental conditions from the perspective of predators.

## Data Availability

This article has no additional data.
